# Biomonitoring of blood cholinesterases and acylpeptide hydrolase activities in rural inhabitants exposed to pesticides in the Coquimbo Region of Chile

**DOI:** 10.1371/journal.pone.0196084

**Published:** 2018-05-02

**Authors:** Muriel Ramírez-Santana, Cristián Farías-Gómez, Liliana Zúñiga-Venegas, Rodrigo Sandoval, Nel Roeleveld, Koos Van der Velden, Paul T. J. Scheepers, Floria Pancetti

**Affiliations:** 1 Department of Public Health, Faculty of Medicine, Universidad Católica del Norte, Coquimbo, Chile; 2 Department of Primary and Community Care, Radboud university medical center, Nijmegen, The Netherlands; 3 Laboratory of Environmental Neurotoxicology, Faculty of Medicine, Universidad Católica del Norte, Coquimbo, Chile; 4 Laboratory of Biomedical Research, Faculty of Medicine, Universidad Católica del Maule, Talca, Chile; 5 Radboud Institute for Health Sciences, Radboud university medical center, Nijmegen, The Netherlands; Weizmann Institute of Science, ISRAEL

## Abstract

In Chile, agriculture is a relevant economic activity and is concomitant with the use of pesticides to improve the yields. Acute intoxications of agricultural workers occur with some frequency and they must be reported to the surveillance system of the Ministry of Health. However the impacts of chronic and environmental pesticide exposure have been less studied. Among pesticides frequently used in Chile for insects control are organophosphates (OP) and carbamates (CB). They are inhibitors of acetylcholinesterase (AChE) and butyrylcholinesterase (BChE). In this study we determined the pattern of both biomarkers activity in three populations with different type of chronic exposure to OP/CB: environmentally exposed (EE), occupationally exposed (OE) and a reference group (RG) without exposure. Besides this, we also measured the activity of acylpeptide hydrolase (APEH), an enzyme involved in relevant functions in the central synapses that is also expressed in erythrocytes and previously reported to be highly inhibited by some OP. A baseline measurement was done in both exposure groups and then a second measurement was done during the spraying season. The RG was measured only once at any time of the year. Our results indicate that people under chronic OP/CB exposure showed an adaptive response through an increase of basal BChE activity. During the spray season only BChE activity was decreased in the EE and OE groups (*p*<0.05 and *p*<0.01, respectively) and the higher magnitude of BChE inhibition was observed in the EE group. The analysis of the frequencies of inhibition above 30% (biological tolerance limit declared by Chilean legislation) indicated that BChE was most frequently inhibited in the EE group (53% of the individuals displayed inhibition) and AChE in the OE group (55% of the individuals displayed AChE inhibition). APEH activity showed the highest frequency of inhibition in the EE group independent of its magnitude (64%). Our results demonstrate that the rural population living nearby agricultural settings displays high levels of environmental exposure. APEH activity seems to be a sensitive biomarker for acute low-level exposure and its usefulness as a routine biomarker must to be explored in future studies. Systematic biomonitoring and health outcomes studies are necessary as well as obtaining the baseline for BChE and AChE activity levels with the aim to improve environmental and occupational health policies in Chile.

## Introduction

Pesticides are used worldwide to increase crop and harvest yields, as well as for domestic and public-health related pest control. Among the chemical classes of these compounds, organophosphorus pesticides (OP) and cabamates (CB) are the most widely used for insect pest control in the agricultural sector [1]. Also known as anticholinesterase pesticides, these compounds inhibit the catalytic activity of acetylcholinesterase (AChE), preventing the breakdown of acetylcholine (ACh) at synapses and leading to overstimulation of muscarinic and nicotinic cholinergic receptors [2,3]. Acute OP exposure and intoxication in humans is of clinical relevance, as it can present with a number of signs and symptoms such as bronchospasms, bronchorrhea, nausea, hypersalivation, bradycardia, sweating, muscle weakness, fasciculations, among others [4]. On the other hand, chronic exposure to low OP doses has also been associated with adverse effects and pathologies such as cognitive impairment [5], inflammation [6], diabetes [7], and cancer [8].

Biomonitoring studies of populations exposed to OP/CB commonly use biomarkers of exposure, effect or susceptibility [1]. For instance, trace amounts of the parent compound in blood, or its metabolites in urine, are used as exposure biomarkers [9] and inhibition of the catalytic activity of erythrocyte AChE is often used as a biomarker of acute OP/CB effects. Erythrocyte AChE, which is equivalent to the enzyme found in cholinergic synapses, is the best proxy to extrapolate OP/CB inhibitory effects at the nervous system synapses [10]. Nevertheless, butyrylcholinesterase (BChE), a plasma enzyme synthesized in the liver with similar catalytic properties to AChE, is also routinely used to determine acute exposure in biomonitoring programs. Reasons to measure BChE instead of AChE activity include its higher degree of reproducibility between laboratories [11,12], and the relative simplicity of isolating plasma from whole blood, wherein the enzyme is found.

AChE and BChE display high interindividual variability in their activities [13], therefore it is necessary to determine a personal baseline measurement prior to the exposure period and to compare the values obtained during or after exposure with this baseline. In fact, Chilean legislation establishes that the biological tolerance limit for the inhibition of AChE or BChE activity for a worker exposed to OPs should not exceed 30% of their personal baseline measurement obtained before exposure [14]. However, this pre-exposure measurement is not usually obtained in exposed populations [15]. Furthermore, there is also no reference value of cholinesterase activity in the Chilean population, which, when added to the high rate of interindividual variability, makes it difficult to interpret biomonitoring results.

The results described herein were derived from a 2011–2014 study geared toward developing a new enzymatic biomarker of cognitive impairment in populations chronically exposed to OP/CB. For this, we recruited volunteers from rural agricultural populations in the Elqui and Limarí valleys of the Coquimbo region of Chile, with more than 5 years of environmental or occupational exposure to pesticides. The proposed biomarker for cognitive impairment was acylpeptide hydrolase (APEH) found in erythrocytes, and AChE and BChE activity levels were used as reference enzymes. Along with attempting to find a correlation between a clinical outcome like cognitive impairment and APEH activity, we also attempted to establish the pattern of enzyme inhibition during spraying. To that end, sampling was performed before and during the spraying season. The detailed description of the study protocol can be obtained in a recent study from our group [16].

APEH (APH, EC 3.4.19.1) is a serine protease with both chymotrypsin-type endopeptidase and *N*-acylpeptide exopeptidase activities [17,18]. It is found in several types of tissue, including the brain [19], muscle [20], erythrocytes [21], and the gastrointestinal tract [22]. Interestingly, APEH has been identified as a highly sensitive direct target for some OPs such as dichlorvos, chlorpyrifosmethyl oxon, and diisopropylfluorophosphate (DFP) [23]. Specifically in brain tissue, it has been demonstrated that APEH is involved in the cellular mechanisms responsible for synaptic plasticity and memory [24,25]. Because of the neuronal implications of APEH, we believed that this enzyme would be a useful marker for chronic exposure associated with cognitive impairment, and/or a biomarker of acute exposure to anticholinesterase pesticides. Thus, we attempted to quantify its activity in erythrocytes, in order to validate its usefulness as a biomarker.

Finally, it is important to highlight that in Chile, there have been few population-based studies regarding pesticide exposure, despite the fact that there are wide populations living and/or working directly or indirectly within the agricultural sector. Two studies have evaluated markers of genotoxicity in temporary female workers in the agricultural sector [26,27]. Other studies have evaluated the neuropsychological effects of OP exposure in children and farm workers [28–30], and one study has assesed paraoxonase-1 (PON1) as a biomarker of OP suceptibility [31]. Nevertheless, there has been no research on ChE inhibition profiles in Chilean populations exposed to OP or CB, neither in the occupational field nor in the general population exposed to environmental pesticide pollution.

## Methods and population

### Study population

**Fig 1:**
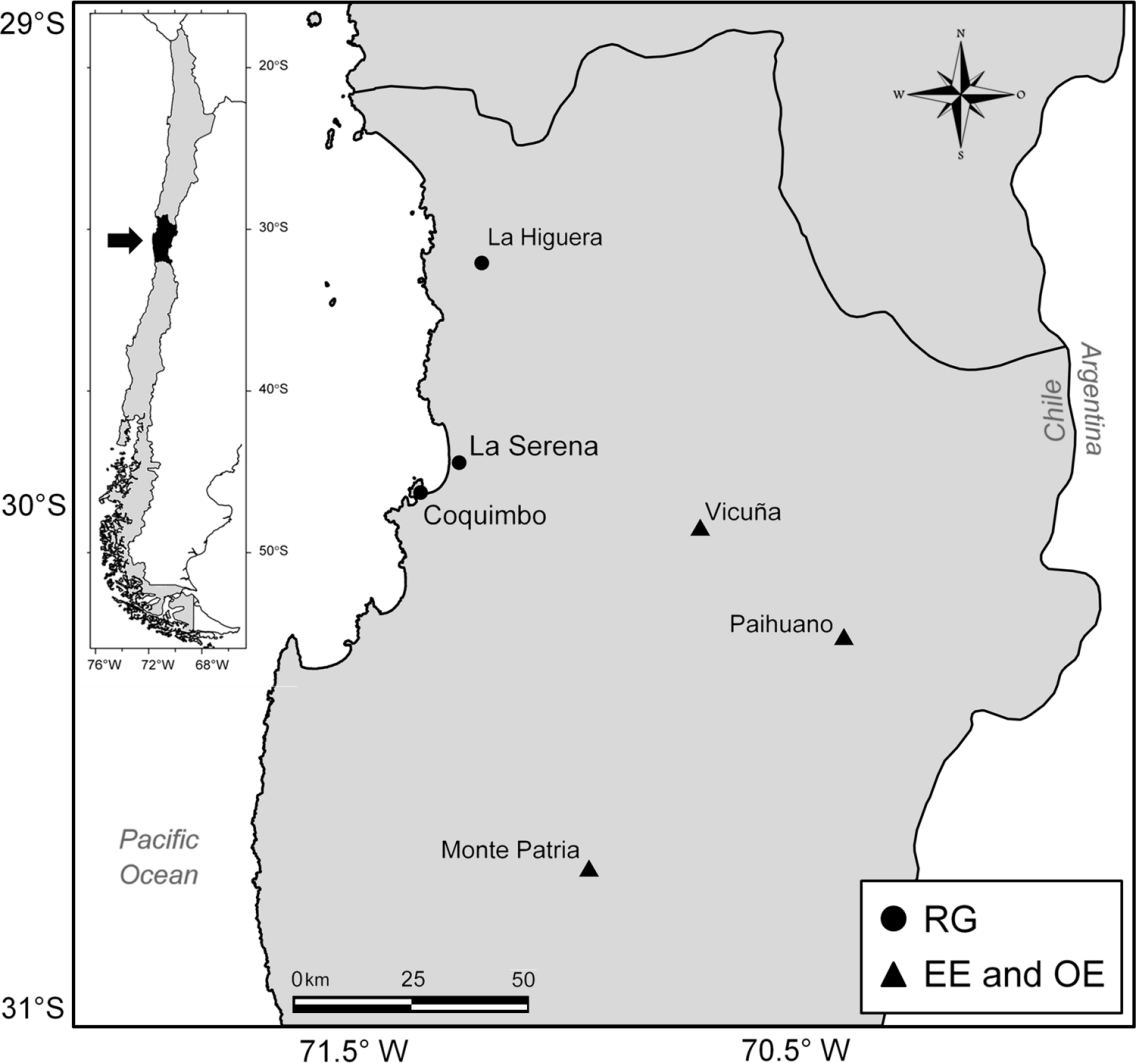
Locations of the Coquimbo Region in Chile from where volunteers were recruited. RG (●) was composed of people living in rural or urban coastal areas. Individuals belonging to EE and OE groups (▲) were recruited from rural areas with intense agricultural activity (Vicuña, Paihuano, Monte Patria).

A mixed study of serial prevalences was conducted by recruiting males and females between the ages of 18 to 50 years old. Three groups were defined *a priori* according to the type of exposure to OP/CB. Sampling was performed by convenience and a questionnaire applied to the volunteers was used for classification in these pre-defined groups: Group 1, the environmentally exposed (EE) group that consisted of individuals living near agricultural land, with no known occupational exposure to OP (*n* = 66); group 2, the occupationally exposed (OE) group that consisted of agricultural workers who reported continuous and direct contact with pesticides for more than 5 years with no episode of acute poisoning (*n* = 87); and group 3, a non-exposed reference group (RG) that consisted of people living in Chilean rural or urban coastal areas, far from agricultural settings and with no known exposure to pesticides (*n* = 100). At least 5 years working with pesticides or living in an area near agricultural settings was required to be included in the EE and OE groups. The study lasted from 2011 to 2014, and annually recruited new volunteers for each study group. EE and OE groups were followed-up over a period of one year in order to cover the pre-spraying season and the spraying season. RG was measured only once at a randomly chosen time during the year. The recruitment zones can be observed in Fig 1. The Coquimbo region is known for fruit production including grapes, avocado, and citrus fruits such as mandarins, oranges, and lemons. A more detailed description of the study design and the inclusion and exclusion criteria can be found in Ramírez-Santana et al. (2015).

### Recruitment and interview

The study design was approved by the ethics committee of the Universidad Católica del Norte in Coquimbo, Chile. After confirmation that the subjects matched the inclusion criteria, individuals were formally recruited by signing an informed consent form that contained detailed information about the study. Recruitment was performed by convenience, and exposure classification was estimated by a questionnaire. In this interview, socio-demographic information, morbidity, and subjective symptoms related to cholinergic syndrome were also obtained. Additionally, individuals belonging to the OE group were asked about the type of agricultural task performed, the use of personal protective equipment, and their pesticide handling training. As a part of the study, all the selected volunteers underwent a neuropsychological evaluation. These results will be published separately.

### Sample collection

Venous blood samples were collected for the quantification of AChE, BChE, and APEH enzyme activity. Sample collection was planned according to the annual agricultural fumigation schedule for grapes and citrus fruits. Under this scheme, EE and OE groups were sampled twice, before and during the fumigation period, with a time lapse of 3 to 4 months. The sampling periods coincided with winter time for the basal measurement (pre-fumigation) and with spring time for the fumigation period. The RG group was sampled only once during the year, since there was no relation to the agricultural schedule.

Sample collection was performed in the field within a mobile laboratory, in order to ensure that the cold chain was maintained. A 3 mL blood sample was collected in vacutainers by venipuncture, using EDTA as an anticoagulant. All samples were processed within 12 hours from the moment of their collection. For this, plasma and cells were separated by centrifugation at 3,000 rpm for 10 min at 4°C. Plasma was isolated from cells and aliquots were distributed in coded tubes. The pellet containing blood cells was washed twice with cold phosphate saline buffer (PBS). Immediately after processing, samples were frozen and stored in liquid nitrogen. Once they arrived at the laboratory, samples were maintained at -80°C until final analysis.

### Quantification of biomarker activities

The origin of the sample was blinded to the technician who performed the biomarker analyses. For AChE and APEH activities, a pellet of blood cells was thawed by keeping it on ice and adding a buffer containing 100 mM Tris-HCl pH 7.4 / 1 mM DTT until the final volume reached 3 mL. Mechanical lysis of cells was accomplished using a tuberculine syringe. Then, the lysate was centrifuged for 30 min at 12,500 *g* at 4°C and the supernatant was aliquoted and frozen for APEH activity measurement. The pellet corresponding to erythrocyte membranes was washed twice with 1 mL of cold 50 mM phosphate buffer, pH 7.9. For each wash, erythrocyte membranes were resuspended and then centrifugued at 12,500 *g* during 30 min at 4°C. After the last wash, the pellet was resuspended in 100 μL of cold 50 mM phosphate buffer, pH 7.9 plus 0.5% triton X-100. AChE activity measurement was performed based on the Ellman’s method [32]. Briefly, the reaction mixture contained 7 μL of the resuspended sample plus 30 μL of 1 mM acetylthiocholine iodide in 1 mL of 50 mM phosphate buffer containing 0.25 mM DTNB. Absorbance was measured every 15 s for 2 min at 406 nm/30°C. The molar extinction coefficient used to calculate the concentration of the final reaction product was ε = 13,600 M^-1^cm^-1^. AChE activity was expressed as specific activity normalized by the concentration of erythrocyte membrane proteins and expressed as mmol x min^-1^ x mg protein^-1^. BChE activity was measured from non-diluted plasma. Next, 7 μL of plasma were used for BChE activity measurement following the same method described for AChE. For this enzyme, specific activity was also expressed as mmol x min^-1^ x mg protein^-1^. The APEH activity from erythrocytes was measured in the cytosolic fraction that corresponds to the supernatant obtained after blood cell lysis and centrifugation. An aliquot of the frozen supernatant was thawed on ice. Then, 5 μL were diluted 1:200 with 0.1 M Tris–HCl, pH 7.4 plus 1 mM DTT. The assay mixture consisted of 25 μL of the diluted cytosolic fraction plus 975 μL 0.1 M Tris–HCl, pH 7.4, supplemented with 1 mM DTT and 20 μL of 0.2 M acetyl-alanyl-*p*-nitroanilide (AANA) as the substrate. The hydrolysis of AANA and *p*-nitroanilide formation (ε_410_ = 8,800 M^-1^x cm^-1^) was measured at 410 nm at 37°C for 40 min and expressed as mmol x min^-1^ x mg Hb^-1^.

Finally, samples of plasma were randomly selected and sent to the Laboratory of Occupational Health of the Instituto de Salud Pública de Chile (ISP). As ISP is the National reference laboratory for this kind of measurement, we compared the BChE activity values obtained by ISP with ours. It should be noted that they do not standarize by protein concentration. In the selected samples, the mean of BChE activity measured at ISP was 4.28 ± 0.81 μmol x min^-1^ x mL^-1^. The mean of the activity measured at our laboratory at Universidad Católica del Norte was 4.34 ± 0.88 μmol x min^-1^ x mL^-1^ (n = 28; *p* = 0.81, Student’s *t*-test). We did not perform a comparison for AChE activity, because the fractionation protocol is different between the laboratories.

### Protein quantification

The protein concentration in samples corresponding to plasma or erythrocyte membranes was quantified using the bicinchoninic acid assay [33].

### Hemoglobin quantification

Hemoglobin concentration present in the supernantants of lysed erythrocytes was measured using the hemoglobin cyanide method [34].

### Data analysis

For sociodemographic characteristics (Table 1), continuous variables were expresed as median and interquatile range and were analized using Kruskal-Wallis test and Mann-Whitney test due to their no-normal distribution, while categorical variables were expresed as frequency and percentages, and analized using independence χ^2^ test.

**Table 1:** Socio-demographic characteristics of the studied groups.

Characteristics	RG (*n* = 100)	EE (*n* = 66)	OE (*n* = 87)
Sex^a^^,^ **			
Female	34 (34%)	41 (62%)	45 (51%)
Male	66 (66%)	25 (38%)	42 (49%)
Age (years)^b^^,^ ***	33 [26–42]	33 [29–38]	38 [32–45]
Education (years of study) ^b^^,^ ***	12 [10–13]	12 [12–14]	10 [8–12]
Smoker^a^^,^ ^ns^			
No	69 (69%)	43 (65%)	55 (63%)
Yes	31 (31%)	23 (35%)	32 (37%)
Alcohol intake (grams/day)^b^^,^ ^ns^	1 [0–4]	1 [0–4]	0 [0–4]
Drugs^a^^,^ ^ns^			
Never tried	79 (79%)	52 (79%)	75 (86%)
Tried sometimes	12 (12%)	11 (17%)	6 (7%)
Frequently used	9 (9%)	3 (5%)	6 (7%)
Environmental exposure (years)^c^^,^ ^ns^	-	20 [10–31]	24 [12–39]
Working exposure (years)	-	-	15 [9–22]

RG = reference group EE = environmentally exposed group, OE = occupationally exposed group. Continuous variables are expressed as median and the interquartile range. Categorical variables are expressed as frequencies and percentage.

^a^Chi-squared test;

^b^Kruskal-Wallis H-test;

^c^Mann-Whitney U-test.

**: *p*<0.01,

***: *p*<0.001,

^ns^: not significant.

**Fig 2:**
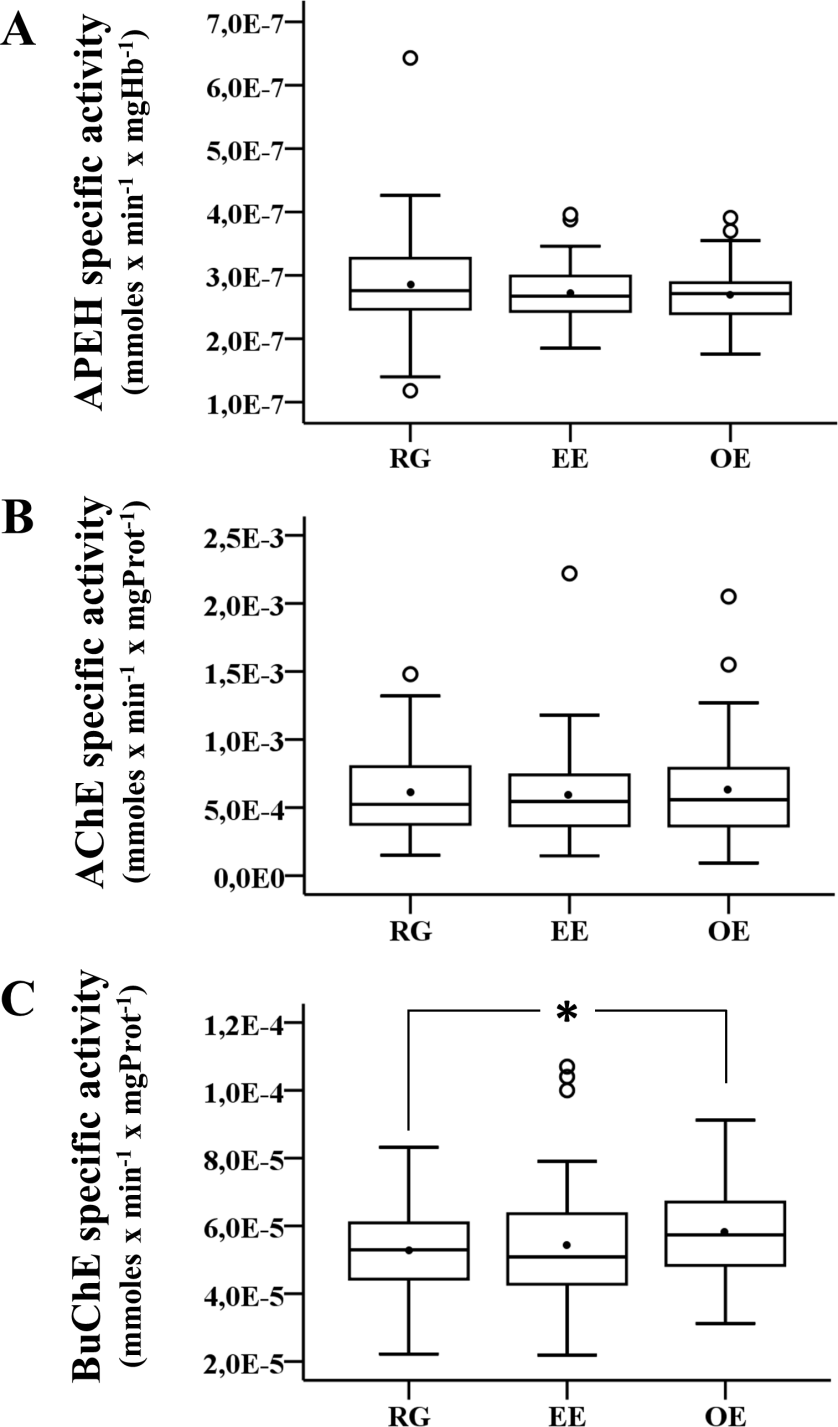
Distribution of enzyme activity levels at baseline. The box plots show the distribution of enzyme activity levels for APEH (A), AChE (B), and BChE (C) for each exposed group before the spray season (baseline). The line inside the box represents the median and the black circle the mean. Statistical analysis for APEH and BChE activities were performed applying ANOVA and for AChE Kruskal Wallis test was used. The arterisk (*) represents the level of significance (*p*<0.05).

The baseline profile of enzyme activity levels (before fumigation) among groups were displayed using boxplot graphics (Fig 2) showing the interquartilic range, the median and the mean. APEH and BChE showed a normal distribution, opposite to AChE, therefore comparisons among groups were done using ANOVA with Tukey’s HSD post-hoc test for APEH and BChE enzimes and Kruskal-Wallis for AChE.

Linear regression analysis was performed to assess the influence of exposure type on basal enzyme activity levels. In this analysis, we included the whole population and made adjustments according to age and gender (Table 2).

**Table 2:** Variables influencing enzyme biomarkers at the baseline measurement (pre-spraying season).

Dependent variable	Explanatory variables	Coefficient			Model		
		Estimative	Standard error	*p*-value	R^2^	Adjusted R^2^	*p*-value
APEH	Exposure group	-8.01E-9	4.36E-9	0.067	0.023	0.011	0.127
	Sex	-6.70E-9	7.35E-9	0.363			
	Age	-4.42E-10	4.25E-10	0.300			
AChE	Exposure group	7.60E-6	2.46E-5	0.758	0.013	0.001	0.347
	Sex	5.77E-5	4.14E-5	0.164			
	Age	2.82E-6	2.40E-6	0.241			
BChE	Exposure group	2.86E-6	1.04E-6	0.006	0.121	0.110	<0.001
	Sex	8.50E-6	1.74E-6	<0.001			
	Age	2.21E-7	1.01E-7	0.030			

Reference explanatory variables: RG = 0; Female = 0

**Fig 3:**
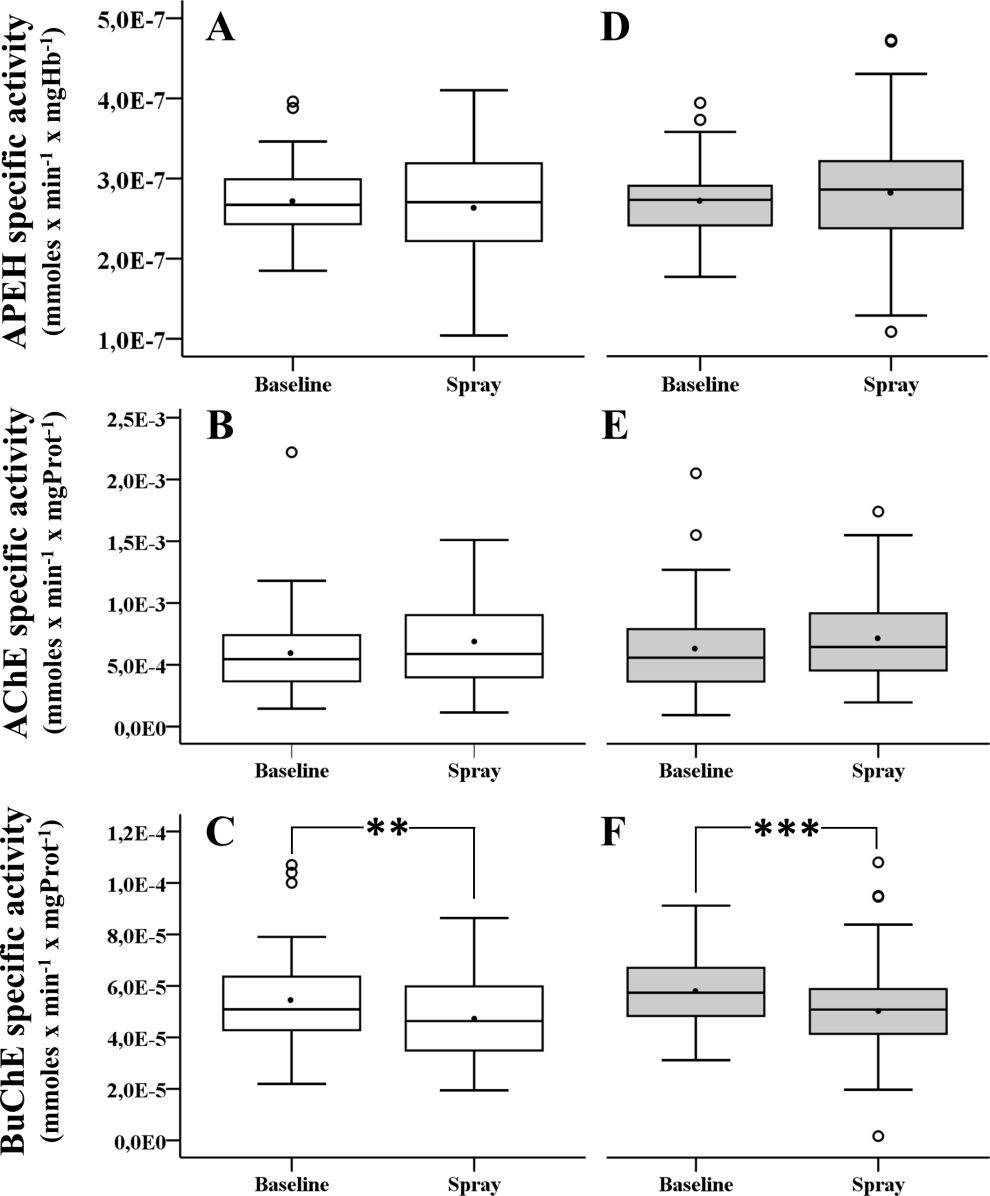
Comparison of enzyme activities before and during the spray season. The box plots show the comparison for APEH (A and D), AChE (B and E), and BChE (C and F) for the EE group (white boxes; n_baseline_ = 66; n_spray_ = 64) and the OE group (grey boxes; n_baseline_ = 87; n_spray_ = 78). The line inside the box represents the median and the black circle the mean. Statistical analysis was performed using the paired *t*-test for APEH and BChE enzymes, and Wilcoxon signed rank test for AChE enzyme, ** *p*<0.01; *** *p*<0.001.

To compare the means of enzyme activity level between baseline and spray season, Student’s *t*-test for paired observations was performed for both exposure groups and for APEH and BChE enzymes, and a Wilcoxon designed rank test for AChE enzyme (Fig 3).

The intra-individual change in enzyme activity from baseline to spray season was calculated as a percentage of change (*I*) by subtracting the enzyme activity observed during the spray season (*A*_*s*_) from the baseline value (*A*_*b*_), and dividing by the baseline value, multiplied by 100. If the enzyme activity was shown to be inhibited during fumigation, this value was positive (see Eq 1).

$$I=\left(\frac{Ab-As}{Ab}\right)\;\times 100\;\%$$ (1)

To compare the frequency and percentage of individuals with some degree of enzyme inhibition, an independent χ^2^ test was used (Table 3).

**Table 3:** Frequency of individuals showing enzyme activity inhibition during spraying.

Exposure group	Percentage of individuals showing inhibition		
	APEH	AChE	BChE
EE (<30% inhibition)	64%	48%	57%
OE (<30% inhibition)	45%	41%	68%
χ^2^*p*-value	**0.02**	0.27	0.13
EE (≥30% inhibition^#^)	14%	25%	30%
OE (≥30% inhibition^#^)	10%	23%	15%
χ^2^*p*-value	0.32	0.45	**0.03**

^#^Percentages were calculated relative to the total of individuals by exposure group. These values represent those individuals above the biological tolerance limit (≥ 30% inhibition).

**Fig 4:**
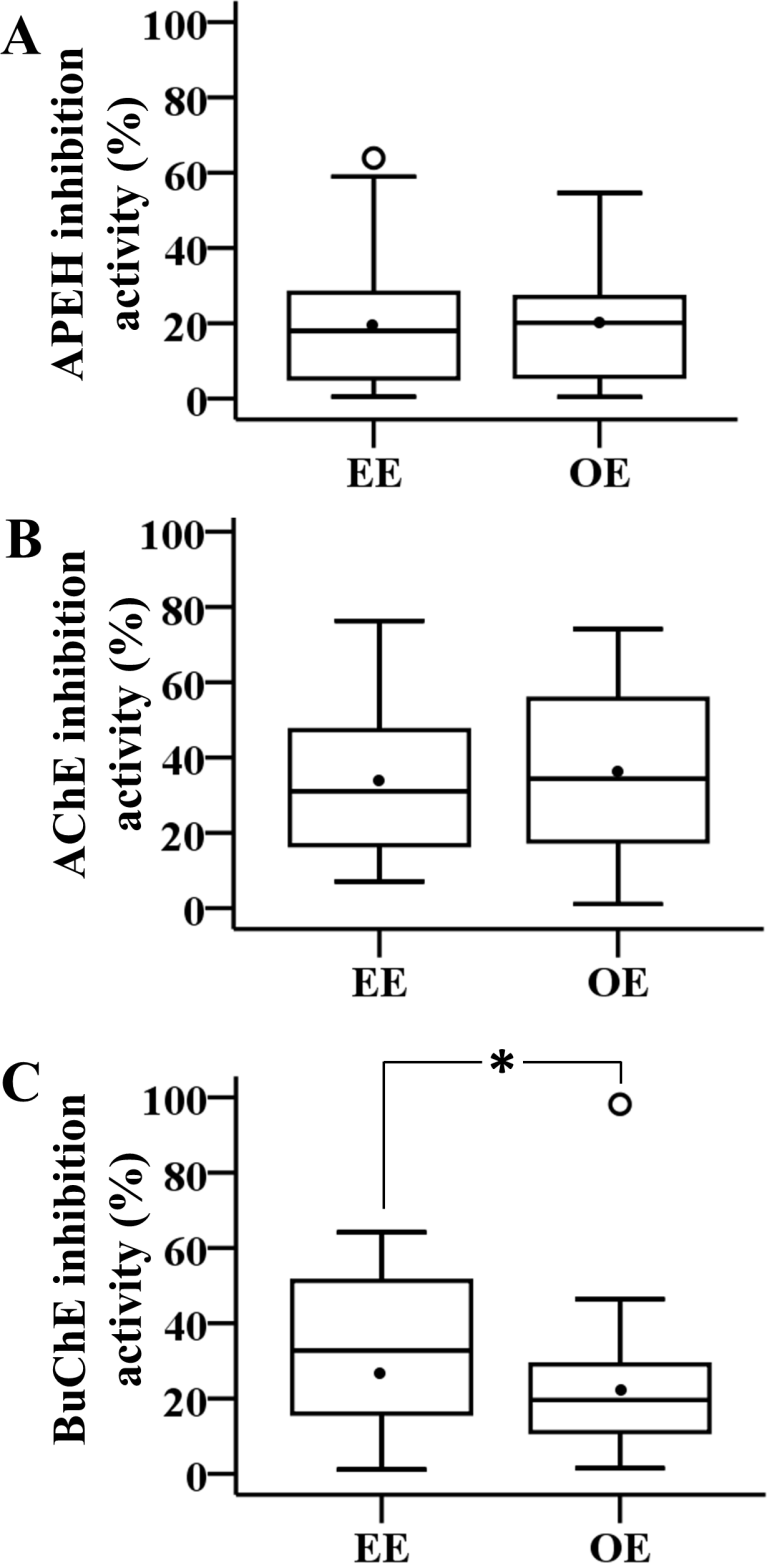
Magnitude of enzyme inhibition during the spray season. The box plots were made considering only those individuals showing enzyme inhibition during fumigation. The panels depict the distribution of the percentages of inhibition of APEH (A), AChE (B) and BChE (C) for the EE and OE groups. The line inside the box represents the median and the black circle the mean. Student *t*-test was used for AChE and BChE activity comparisons, and Mann-Whitney test for APEH, *p<0.05.

To compare the magnitude of inhibition of the three enzymes among groups, Student’s *t*-test was used for AChE and BChE activities comparisons between EE and OE groups, and Mann-Whitney test for APEH activity (Fig 4).

Finally, taking into account that only BChE activity showed significant inhibition in both exposed groups (EE and OE) (Fig 3), linear regression analysis was only performed for this enzyme to assess the influence of the exposure group on its inhibition. For this, we included all individuals where BChE inhibition was observed, and adjusted for age and gender (Table 4).

**Table 4:** Variables influencing BChE inhibition.

Dependent variable	Explanatory variables	Coefficient			Model		
		β	Standard error	*p*-value	R^2^	Adjusted R^2^	*p*-value
BChE	Exposure group	-9.86	3.91	0.014	0.087	0.055	0.050
	Sex	4.39	3.85	0.257			
	Age	-0.04	0.23	0.875			

Reference explanatory variables: EE = 0; Female = 0

Data were analyzed using SPSS software, version 22 (SPSS Inc. Chicago, IL). A *p* value of <0.05 was used as the criterion for statistical significance.

## Results

### Population characteristics

Three populations with different types of OP/CB exposure were recruited, and their socio-demographic characteristics are presented in Table 1. Significant differences in sex, age, and years of education were observed. Sex was distributed differently among groups (*p*<0.01); the female population was higher in the EE and OE groups compared to the RG group. Individuals belonging to the OE group were older (5 years on average) and had received fewer years of education (*p*<0.001). Other confounding factors such as smoking, alcohol intake, or sporadic drug consumption did not show any significant differences among groups. Both the EE and OE groups reported having lived many years in a rural environment close to agricultural activity (average of 20 years). Workers from the exposed OE group reported having worked in contact with pesticides for approximately 16 years.

### Enzyme activity profiles of the exposure groups at baseline

Fig 2 shows the distribution of enzyme activity levels according to the exposure group at baseline (before spraying). The mean of BChE activity at baseline was higher in the OE group compared to the RG group (2.8 x 10^−7^±7.4 x 10^−8^ mmol x min^-1^ x mg^-1^ and 2.7 x 10^−7^±4.4 x 10^−8^ mmol x min^-1^ x mg^-1^, respectively; *p*<0.05). APEH and AChE activities were not statistically different among groups.

### Analysis of the variables influencing enzyme activity at baseline

To evaluate the influence of exposure type on the activity of each enzyme, we performed a linear regression analysis considering the whole population, adjusted by confounders such as age and sex. Table 2 shows that for APEH and AChE activity levels at baseline, the exposure group, sex, and age did not explain the variability observed. BChE activity, on the other hand, was mainly influenced by exposure group, when adjusted for age and sex. Moreover, BChE activity was higher in males than females (5.9 x 10^−5^±1.5 x 10^−5^ mmol x min^-1^ x mg^-1^ for males and 5.1 x 10^−5^±1.3 x 10^−5^ mmol x min^-1^ x mg^-1^ for females; *p*<0.001) and, as indicated by the linear regression analysis, its activity increased with age.

### Influence of temporality on enzyme activity profiles of the EE and OE exposure groups

The effect of temporality on enzyme activities is shown in Fig 3 for both exposure groups. In charts A, B, and C, the activity distribution of the three enzymes (APEH, AChE, and BChE, respectively) is shown for the EE group, before and during spraying. A decrease in enzyme activity during spraying (4.7 x 10^−5^ ± 1.6 x 10^−5^ mmol x min^-1^ x mg^-1^; mean ± SD), compared to the baseline value (5.5 x 10^−5^ ± 1.7 x 10^−5^ mmol x min^-1^ x mg^-1^; *p* = 0.007), was observed only for BChE. In charts D, E, and F, the distribution of enzyme activity levels for the OE group at both time points is presented. As before, in the OE group, only BChE activity showed a significant decrease during spraying (5.8 x 10^−5^±1.4 x 10^−5^ mmol x min^-1^ x mg^-1^ at baseline, and 5 x 10^−5^±1.7 x 10^−5^ mmol x min^-1^ x mg^-1^ during the spray season; *p* = 0.002).

### Analysis of the magnitude and frequency of inhibition for the three biomarkers in both exposure groups

The percentage of inhibition of each enzyme was calculated using Eq 1. Table 3 (upper panel) shows the frequency of individuals who exhibited some degree of inhibition for the three enzymes in both exposure groups. Although we did not observe significant differences in the frequency of individuals showing BChE or AChE inhibition in either group, more than half of the total volunteers displayed BChE inhibition. This included 57% of the EE group, and 68% of the OE group. On the other hand, we found a significant difference in the frequency of individuals with inhibition of APEH activity, with a higher frequency of inhibition in the EE group compared to the OE group (64% versus 45%, *p* = 0.02, χ^2^ test).

The lower panel of Table 3 shows the frequency of inhibition above the biological tolerance limit for cholinesterases as established by Chilean legislation. Only BChE levels were significantly different in regard to the frequency of individuals with greater than or equal to 30% inhibition between groups. It is worrying to note that 30% of individuals from the EE group demonstrated levels above the established limit of biological tolerance. This contrasts with the frequency of individuals from the OE group, where only 15% of the group demonstrated cholinesterase inhibition levels above the biological tolerance limit (*p* = 0.03, χ^2^ test).

Fig 4 demonstrates only those individuals in whom inhibition of enzyme activity was observed, regardless of magnitude. Only BChE inhibition levels were significantly different between both exposure groups, being more inhibited in the EE group compared to OE group (32.4% ± 19% in EE *versus* 22.5% ± 16.4 in OE, *p*<0.01, Student’s *t*-test).

### Analysis of the variables influencing BChE inhibition

As BChE was the only biomarker that was significantly inhibited in the EE and OE groups during fumigation, we wanted to determine the variables that modulate its inhibition using a linear regression analysis. Table 4 shows that the type of exposure, adjusted by sex and age, contributes to BChE inhibition. In general, a higher magnitude of BChE inhibition is observed in the EE group compared to the OE group. Males appear to be more prone to BChE inhibition than females, and BChE inhibition appeared to be negatively modulated by age, although none of those variables are significant in the model. Only the type of exposure was significant.

## Discussion

This study represents the first effort to conduct systematic biomonitoring of blood cholinesterases in individuals occupationally and environmentally exposed to OP/CB pesticides in rural locations of Chile. This study also measured erythrocyte APEH activity in these populations. A key finding is that the general population living near agricultural lands, represented by the EE group, displayed significant inhibition of BChE activity during the spray season, reflecting a condition of environmental exposure to OP/CB pesticides. Our results also indicate that APEH activity is not a sensitive biomarker that reflects either basal chronic exposure, or acute inhibition above the biological tolerance limit enforced by Chilean legislation. However, the frequency of individuals showing some degree of inhibition is better detected by this biomarker in environmentally exposed populations.

It is important to mention that although biomonitoring of urinary metabolites of pesticides is considered the gold standard for demonstrating exposure in human populations [35–37], in this study the analysis of cholinesterase inhibition patterns in both exposure groups also served as an estimate of the degree of exposure to OP/CB pesticides [38–40].

Considering the frequency of inhibition of the three enzyme biomarkers measured, we observed that in the EE group, 64% of the volunteers displayed some degree of APEH inhibition, while 57% showed BChE inhibition, and 48% demonstrated AChE inhibition (Table 4). Because APEH activity was inhibited in a high proportion of rural village inhabitants during spraying, APEH activity is a good candidate for a putative sensor that may be highly sensitive to changes in the environmental pesticide burden. However, when we compare the average of APEH activity in the whole EE group (not only in the individuals showing inhibition of this enzyme) before and during fumigation, there were no significant differences (see Fig 3A). These differences only become significant when we compare BChE activity before and during fumigation in the EE group. In other words, in terms of magnitude of inhibition, BChE inhibition was not only observed in more than 50% of individuals, but also displayed a higher inhibition magnitude on average (see Fig 3C). Regarding the OE group, BChE activity is again the biomarker that displays the higher frequency of inhibition, with 68% of individuals showing some degree of inhibition. AChE and APEH were inhibited in 41% and 45% of the population, respectively (Table 4). Moreover, as with the EE group, BChE is the biomarker that, on average, is more significantly inhibited during fumigation in the whole OE group (see Fig 3F). This finding is not surprising, considering that BChE is a sensitive biomarker of exposure to certain OP/CB pesticides, especially at low levels of exposure [41]. Among the OP/CB that display a higher affinity toward BChE over AChE is chlorpyrifos oxon, the toxic metabolite of chlorpyrifos that possesses an inhibitory constant three orders of magnitude lower for BChE compared to AChE [42]. In fact, it has been reported that oral doses of chlorpyrifos given to volunteers inhibited plasma BChE activity in 85% without affecting erythrocyte AChE activity. As no signs of toxicity were observed in the study, the inhibition of BChE activity is not considered an endpoint for risk assessment [43–45]. Thus, in our study we measured neuropsychological performance as an outcome. The complete report of these findings will be published separately; however, it is important to mention that we found memory and executive function impairment in individuals belonging to the EE group with BChE inhibition above the biological tolerance limit (30%) (manuscript in preparation). Our laboratory has also published evidence that supports this observation. In a pilot study in which a small sample was recruited in the same rural locations as those reported in the present study, we found deficits in the same cognitive areas in environmentally exposed volunteers [30]. From the analysis presented here, the high percentage of inhibition observed in the EE group is worrying. When we analyze the distribution profile of participants that demonstrated over 30% of inhibition for the three biomarkers in the EE group, we found that 30% of the individuals showed BChE inhibition over the biological tolerance limit, followed by 25% for AChE inhibition, and 14% for APEH inhibition. In the OE group, these values were 15%, 23%, and 10% for BChE, AChE and APEH, respectively. These percentages indicate that more people were at risk within the general rural population (EE group) than among agricultural workers (OE group) at the instant that the sampling was performed. This suggests that agricultural use of OP leads to higher internal exposure in the rural population compared to levels of occupational exposure in agricultural workers who use personal protective equipment (see S1 Table). Nevertheless, it is necessary to determine if factors that influence the amount of exposure in the OE group, like individual working practices or the use of protective equipment, have an influence on biomarkers activities [46]. This information was obtained from the survey completed by volunteers classified in the OE group and will be presented and discussed in a separate publication regarding policy. In general, there are many other factors influencing the degree of exposure in the EE and OE groups. Among these are the types of OP/CBs reaching the exposure pathways in one group or another, the intra-individual susceptibility that relies on enzyme polymorphisms or other factors, and specifically for the EE group, in climate and geographical aspects that have an impact on the environmental spill out of pesticides. Regarding the type of OP/CB to which people were exposed, we did not include this question in the survey. However, the volunteers performed their agricultural work on farms where grapes, oranges, lemons, and other crops are grown, and it is known that citrus fruits are fumigated mainly with chlorpyrifos in the months of December and January (spring/summer) to control different types of insects, and with fenamiphos and ethoprophos for the control of nematodes [47]. On the other hand, grapes are fumigated with chlorpyrifos, dimethoate, diazinon, profenofos and azinfos methyl during the spring months[48]. To complement this information, among the insecticides most frequently sold in the locations where the study was carried out are oxamyl (a carbamate) and chlorpyrifos (organophosphate), and among the fungicides, sulfur and mancozeb (dithiocarbamate) [49].

In baseline measurements, we found significant differences in average BChE activity levels between the RG and OE groups (Fig 2C), with increased activity in the OE group. A similar observation was previously reported in green-house workers exposed to low-levels of pesticides who displayed higher levels of BChE activity in the low exposure period. Also, these individuals showed enhanced serum PON1activity. It is hypothesized that an up-regulation of these enzymes could occur as an adaptive response to compensate for the adverse effects of pesticide exposure [50]. Interestingly, such up-regulation, along with AChE inhibition, have both been observed in populations exposed to non-organophosphates pesticides like pyrethroids or neonicotinoids, among others. These authors argued that AChE inhibition could be an indirect effect of lipid peroxidation at cell membranes under oxidative stress [51]. Certainly, within the complexity of the factors that could be crucial for health impairments in populations chronically exposed to pesticides, it should be also considered that OP/CB molecules could exert its effect through other toxicological targets different from cholinesterases [6,52,53]. One of these non-cholinesterase targets is APEH, which is inhibited by some but not all OP pesticides. Moreover, there is compelling evidence that points to a relevant function of this enzyme in detoxification pathways of oxidized protein substrates, justifying its activity measurement in blood samples of individuals chronically exposed to pesticides. The results obtained do not show changes in its exopeptidase activity among exposure groups at baseline (Fig 2A) or in EE or OE groups during spraying (Fig 3A and 3D). However, it is known that, in erythrocytes, APEH also behaves as an endopeptidase towards oxidized peptides [21,54–56]. Therefore, a problem that should be addressed in future studies is to determine the profile of the endopeptidase activity instead of the exopeptidase activity of APEH. It is important to keep in mind that the ultimate reason to measure APEH activity in our study was to determine if it serves as a biomarker of cognitive impairment in individuals chronically exposed to pesticides. The results showing such associations will be published soon. However, it is important to mention that exo- and endopeptidase activities of APEH are decreased in blood samples of Alzheimer’s patients [57]. The relationship between chronic exposure to pesticides, cognitive impairment or neurodegenerative diseases, and the role of APEH activity as an early biomarker of this pathological progression, remains to be elucidated.

This study has limitations that could be remedied with future research. For example, we did not include the determination of the environmental burden of pesticides, or try to assess internal OP/CB exposure through urinary metabolite levels. OP exposure status was verified using sociodemographic data, obtained using a questionnaire. This does not allow for an accurate classification of our sample according to the degree of OP exposure, which may have resulted in exposure misclassification. We also assumed that environmental exposure via dietary intake of OP residues from fruits and vegetables would make a similar contribution in all the studied groups. Another limitation is that age and sex were not homogeneous between groups. Other confounders, such as smoking, alcohol consumption, and drug use, did not show significant differences. Therefore, the analysis in this report considered sex and age as confounders, which has been previously described in the literature [58–60].

This study is the first reported evidence from Chile of the high degree of exposure in the general population living nearby agricultural lands, where pesticide spraying is performed to improve yields. The results suggest that systematic biomonitoring and health outcome studies are necessary in these populations, including procurement of data on baseline BChE and AChE activity levels, with the aim to improve environmental and occupational health policies in Chile.

## Supporting information

S1 File Database. The excel file contains the sociodemographic information and enzyme activities reported in the article.
(XLSX)

S1 Table Use of personal protection equipment in occupational exposed individuals. Individuals belonging to the OE group were asked about the use of personal protection equipment in the questionnaire. Fifty-four percent of the individuals declared to use incomplete protection or no-protection equipment at all.
(DOCX)
