# Supplementary material for: Biomonitoring of blood cholinesterases and acylpeptide hydrolase activities in rural inhabitants exposed to pesticides in the Coquimbo Region of Chile
Source: PLoS One. 2018 May 2;13(5):e0196084. doi: 10.1371/journal.pone.0196084 (PMC5931667; doi:10.1371/journal.pone.0196084)
Supplement: S1 Table — Individuals belonging to the OE group were asked about the use of personal protection equipment in the questionnaire. Fifty-four percent of the individuals declared to use incomplete protection or no-protection equipment at all. (DOCX) [file pone.0196084.s002.docx]

| **S1 Table**  **Use of personal protection equipment in occupational exposed individuals (n=87)** | | |
| --- | --- | --- |
| **Protection Equipment** | **Number** | **Percentage** |
| Mask with filter, gloves and eyes protection | 36 | 41% |
| Incomplete protection | 32 | 37% |
| No protection | 15 | 17% |
| No answer | 4 | 5% |
| Total | 87 | 100% |
